# Determinants of the Public Health Promotion Behavior: Evidence from Repurchasing Health Foods for Improving Gastrointestinal Tract Functions

**DOI:** 10.3390/ijerph17207604

**Published:** 2020-10-19

**Authors:** Ku-Yuan Lee, Chien-Yu Wei, Min-Hua Wu, Chi-Ming Hsieh

**Affiliations:** 1 College of Intelligence, National Taichung University of Science and Technology, No.129, Sec.3, Sanmin Rd, North Dist., Taichung City 40401, Taiwan; howkaka@hotmail.com; 2 Graduate Institute of Bio-Industry Management, National Chung Hsing University, Taichung City 40227, Taiwan; justyu407@gmail.com; 3 Institute of Food Safety Management, National Pingtung University of Science and Technology, 1, Shuefu Road, Neipu, Pingtung 91201, Taiwan; 4 International Bachelor Program of Agribusiness, National Chung Hsing University, 145 Xingda Rd., South Dist., Taichung City 40227, Taiwan

**Keywords:** health food, health belief model, perceived behavioral control, gastrointestinal functions

## Abstract

Researchers believe that health foods can promote health and that the consumption of health foods can effectively help people to maintain their health. This study mainly adopted the health belief model (HBM) integrated with perceived behavioral control to investigate the repurchase behavior of consumers with regard to health foods that improve gastrointestinal functions. We obtained 550 valid questionnaires from consumers who had purchased gastrointestinal health foods and conducted structural equation modeling. Results from our analysis revealed that perceived susceptibility, perceived severity, perceived benefits of action, and perceived behavioral control exert a positive influence on repurchase intention and that perceived barriers of action exerts a negative influence on repurchase intention. Furthermore, repurchase intention was found to have a positive impact on repurchase behavior. The results of this study can be used as a reference for health food marketing strategies and public health behavior promotions.

## 1. Introduction

The mortality rate in Taiwan decreased by an average of 1.6% annually in the past decade compared to WHO’s age-standardized mortality rate (408.2 deaths per 100,000 population in 2000). There was a gradual decline in the mortality rate in Taiwan every year [1]. However, as the mortality rate declined, the structure of the population also changed. At the end of March 2018, the senior population in Taiwan exceeded 14%, the threshold in which a country is deemed an aged society. This transition into an aged society was prompted by advances in medical technology and rising awareness of preventive medicine, health preservation, and healthy living and consumption. For instance, approximately 30–40% of cancers can be prevented through early diet intervention; some diseases could be prevented by the intake of specific nutrients. In the treatment of diseases, health foods and medicine play different roles; the former is more closely classified as preventive medicine. In the long-term scheme of medical matters and public health, the consumption of health foods can reduce costs and thereby improve overall health and quality of life.

The purpose of gastrointestinal health foods is to improve gastrointestinal function. According to the Ministry of Health and Welfare [1], the second and third of the five leading causes of cancer death involve the gastrointestinal tract, highlighting the crucial need for the public to understand the importance of gastrointestinal health. Thus, health foods aimed at improving gastrointestinal functions could be conducive to improvements in human health. Unlike medicine, health foods cannot treat diseases, but they can promote overall health. They can only serve as a daily means of promoting health and preventing diseases, and without continued consumption, they lose these effects. As the consumption of health foods increases nutritional intake, promotes health, or slows aging, the act of purchasing health foods can be regarded as a health behavior. Thus, prompting consumers to repurchase or continue purchasing health foods is critical to maintaining their health-promoting effects.

The health belief model (HBM) proposed by Rosenstock [2] has been widely used to predict behavior associated with health and disease prevention. Many studies also have demonstrated that the HBM can effectively explain and predict health behavior. However, applying the HBM to health foods or products has been less common [3], indicating that the application of HBM needs more exploration in other contexts such as health foods. Past research [4] pointed out that many other factors besides health beliefs (such as past experiences and habits, non-health-related factors, economic factors, and environmental factors) exert a decisive impact on health behavior. Achterberg and Miller [5] further claimed that, to explain the changes in human diet and nutrition behavior, incorporating other theories or dimensions regarding individual behavior may be more effective. Thus, while exploring the factors influencing repurchase behavior involving gastrointestinal health foods, we also integrated the dimension of perceived behavioral control to construct the theoretical framework, predicting the purchase behaviors involving gastrointestinal health foods.

## 2. Literature Review

### 2.1. Health Foods Improving Gastrointestinal Functions

The Food Industry Research and Development Institute [6] stated that, based on its investigation, the scope of the health food market in Taiwan in 2017 was Taiwan Dollar (TWD) 121.4 billion and the health food market had an annual growth rate of over 5%. In 2017, there were more than 315 health food items, and the number has continued to rise. In terms of function, the Information of Quality Control (IQC) Product Safety Database pointed out that out of all the various categories, health foods regulating lipids was the largest category, containing 159 items. This was followed by health foods improving gastrointestinal functions, with 81 items, and immunomodulatory health foods, with 46 items. This indicates that health foods improving gastrointestinal functions are important products for manufacturers and consumers. The methods to evaluate the effectiveness of health foods in improving gastrointestinal functions [7] indicate that any health foods that can aid digestion and absorption, improve intestinal bacterial flora, promote gastrointestinal motility, or help the intestines to maintain normal function can be regarded as effective in improving gastrointestinal functions. As presented in June 2018, there were 82 items that qualified as gastrointestinal health foods.

### 2.2. Health Belief Model (HBM)

In the early 1950s in the US, the public had lukewarm responses to the free or low-cost disease prevention screening program launched by the US Public Health Service [8]. At the time, few researchers were studying behaviors associated with health problems, and very little literature on health behavior existed. In view of this, researchers including Hochbaum, Leventhal, Kegeles, and Rosenstock utilized the concepts of field theory and integrated one another’s research to develop a theoretical model that could explain the behaviors of individuals regarding preventive health: The Health Belief Model (HBM).

The HBM can be divided into three parts: individual perceptions, modifying factors, and likelihood of action.

#### 2.2.1. Individual Perceptions

Individual perceptions can be further divided into perceived susceptibility and perceived severity.

(1)  Perceived susceptibility:

Perceived susceptibility refers to an individual’s subjective perception of the risk of themselves contracting a disease, of a certain group contracting a disease, or of themselves contracting a disease due to a certain behavior or trait. In their study on the sexual behavior and HIV/AIDS prevention measures of African immigrants in the US, Asare and Sharma [9] found that perceived susceptibility can be used to predict HIV/AIDS-related health behavior.

Based on the inferences above, we formulated the following research hypothesis:

**Hypothesis 1** **(H1).** Perceived susceptibility exerts a positive influence on repurchase intention.

(2)  Perceived severity:

Perceived severity refers to an individual’s perception of the severity of contracting a disease, including assessments of its clinical and social consequences. In their study on mammography screening, Schwartz et al. [10] observed that subjects perceiving greater severity were more likely to take health actions.

Based on the inferences above, we formulated the following research hypothesis:

**Hypothesis 2** **(H2).** Perceived severity exerts a positive influence on repurchase intention.

#### 2.2.2. Estimated Effects of an Individual Taking Action

The estimated effects of an individual taking action can be further divided into perceived benefits of action and perceived barriers of action.

(1)  Perceived benefits of action:

Perceived benefits of action refers to an individual’s subjective belief that an action will be able to effectively prevent or predict the threat of a disease or mitigate the adverse consequences of contracting a disease. Rodriguez-Reimann et al. [11] investigated the reasons behind various behaviors regarding tuberculosis prevention of Mexican Americans of different genders and observed that when an individual perceives greater benefits from an action, they will be more likely to take said health action. This means that perceived benefits of action has a positive impact on health behavior.

Based on the inferences above, we formulated the following research hypothesis:

**Hypothesis 3** **(H3).** Perceived benefits of action has a positive influence on repurchase intention.

(2)  Perceived barriers of action:

Perceived barriers of action refers to an individual’s assessment of the obstacles that they will encounter while taking an action, obstacles that include tangible and intangible costs. Wai et al. [12] examined the unwillingness of hepatitis B patients to undergo screening tests and found that perceived barriers of action, such as overly long wait times, can reduce the willingness of patients to take health actions.

Based on the inferences above, we formulated the following research hypothesis:

**Hypothesis 4** **(H4).** Perceived barriers of action has a negative influence on repurchase intention.

#### 2.2.3. Cues to Action

Cues to action refers to the sources of strategies and references that promote preventive behavior and include internal and external cues. Internal cues include an individual’s perception of their own health or discomfort, while external cues include information from mass media, advice from friends and family, and reminders from medical personnel. Chen and Tseng [13] investigated the willingness of senior and vocational high school students in Tainan City to consume health foods and observed that cues to action exerts a positive impact on willingness to consume health foods.

Based on the inferences above, we formulated the following research hypothesis:

**Hypothesis 5** **(H5).** Cues to action has a positive influence on repurchase intention.

**Figure 1:**
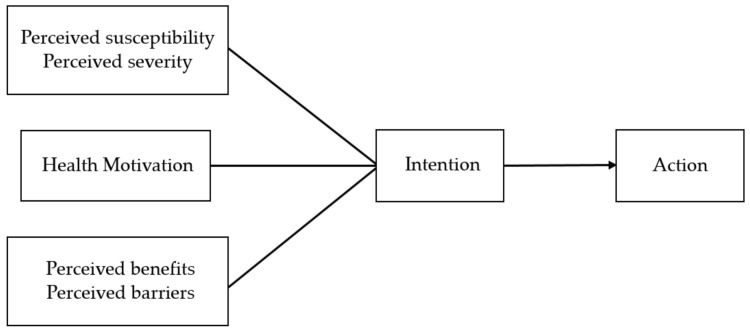
Framework of the Health Belief Model with integrated concepts.

Just as Rosenstock divided the variables of the HBM into three categories, Sheeran and Abraham [14] combined perceived susceptibility and perceived severity into a major concept called threat perception, merged perceived benefits of action and perceived barriers of action into another major concept called behavioral evaluation, and kept cues to action as a major concept by itself. Threat perception, behavioral evaluation, and cues to action jointly influence behavioral intention (motivation) and have good predictive effects. Based on these concepts, Wang reconstructed the framework of the HBM (as shown in Figure 1).

Perceived behavioral control consists of self-efficacy and facilitating conditions and refers to the difficulty that an individual perceives in completing a certain action. Perceived behavioral control refers to the difficulty that an individual perceives in completing a certain action. This variable was created to fill the gaps of the theory of reasoned action (TRA) and represents potential involuntary factors. Taylor and Todd [15] deconstructed perceived behavioral control into two parts: self-efficacy and facilitating conditions. The perceived barriers in the HBM measure the difficulty of behavior in a negative direction. Meanwhile, the self-efficacy and facilitating conditions measure the difficulty of behavior in a positive direction.

Bandura [16] defined self-efficacy as an individual’s judgment of their own ability to execute a certain action and defined facilitating conditions as the resources and assistance that an individual receives when executing a certain action, such as time and money. In an investigation on factors that influence behavioral intentions regarding food consumption, Lin and Feng [17] found that perceived behavioral control exerts a positive impact on consumer behavior and that it is one of the primary influencing factors.

Based on the inferences above, we formulated the following research hypothesis:

**Hypothesis 6** **(H6).** Perceived behavioral control has a positive influence on repurchase intention.

In health behavior research, the HBM has been regarded as a mature theoretical framework. Many past studies on health behavior have verified the effectiveness of the HBM in predicting and explaining health-related behavior. However, the majority of these studies only applied one of these theories. This study focused on the repurchase behavior involving health foods that improve gastrointestinal functions and regarded the purchase of gastrointestinal health foods as a health behavior. We therefore constructed our research framework based on the HBM with the perceived behavioral control concept to enhance its completeness.

Accordingly, we also assumed that repurchase intention exerts a positive and significant impact on repurchase behavior:

**Hypothesis 7** **(H7).** Repurchase intention has a positive influence on repurchase behavior.

**Figure 2:**
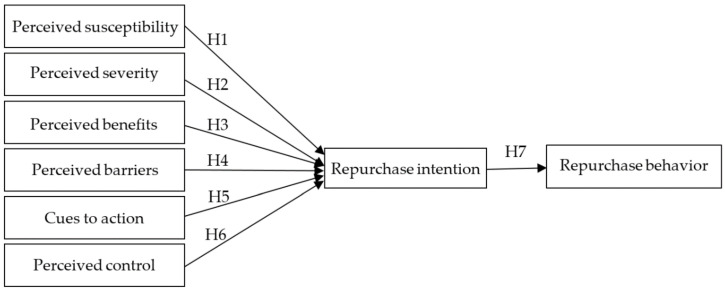
Research framework.

Based on the above discussion, the hypothesized relationships based on the HBM among eight constructs using a structural equation modeling approach are presented in Figure 2.

## 3. Methodology

### 3.1. Measurement Scale Development

This study established the measurement scales including all constructs and 34 corresponding items based on prior studies as presented in Table 1. Each question item was measured on a seven-point Likert scale with 1–7 points respectively given to the following responses: strongly disagree, disagree, somewhat disagree, neutral, somewhat agree, agree, and strongly agree. A higher score indicated a higher degree of agreement. The demographic variables in this study included gender, age, occupation, educational background, place of residence, personal average monthly income, monthly budget willing to spend on gastrointestinal health foods, monthly budget willing to spend on health promotion, frequency of health checkups, and whether any friends and family members had gastrointestinal diseases.

### 3.2. Data Collection Procedures and Analysis Approaches

Data collection was mainly through one primary stage that corresponded with the design of the questionnaire. This study targeted people who had experiences of purchasing health food products to improve their gastrointestinal functions. Thus, this study adopted an online survey with snowball sampling to access specific populations who had experiences of consuming health food products to avoid gastrointestinal disorders. Participants were also encouraged to assist in distributing to relatives and friends who had eaten gastrointestinal health products. In addition, the questionnaires were distributed to the primary forums of the Pi Ti Ti (PTT) Bulletin Board System (BBS), which is the largest terminal-based BBS based in Taiwan, and groups related to health foods in Facebook. All subjects were informed in advance that they would receive an incentive to increase the participation rate. Previous studies suggested a ratio of sample size to the number of observed variables ranging from 1:10 [24] to 1:20 [25]. This study was conducted with 34 items within eight constructs—perceived susceptibility, perceived severity, perceived benefits, perceived barriers, cues to action, perceived control, repurchase intention, and repurchase behavior. A sufficient sample size of at least 340 observations should be sufficient for manipulating the measurement model or exploratory factor analysis (EFA).

After eliminating invalid questionnaires using screening questions, inverse screening questions, and erroneous responses, we obtained a total of 550 valid questionnaires. The overall response rate was 80.6%. To examine the reliability, validity, and structural equation modeling (SEM) of the measurement scales, collected data were entered and analyzed in the SPSS (Statistical Package for the Social Science) version 23.0 (IBM, New York, NY, USA) for Windows, and Amos (Analysis of Moment Structures) version 22.0 (IBM, New York, NY, USA) for Windows.

## 4. Results

### 4.1. Descriptive Statistics Analysis

The descriptive statistics and reliability of the questionnaire results were analyzed using SPSS Statistics 23.0, and the validity analysis and structural equation modeling were conducted using IBM Amos 22.0.

We recovered a total of 550 valid questionnaires. In terms of gender, 62.8% of the respondents were female. In terms of age, the largest and second largest groups comprised individuals between the ages of 21 and 30 years and between the ages of 31 and 40 years, respectively, accounting for 35.6% and 21.7% of the respondents, respectively. For marital status, 64.5% of the respondents were single. For occupation, the largest and second largest groups were students and workers in the service industry, respectively occupying 21.7% and 17.0% of the respondents. In terms of educational background, the largest group had attained a bachelor’s degree as the highest qualification, accounting for 62.8% of the respondents. Regarding place of residence, the majority (74.3%) lived in one of the six major cities in Taiwan. For personal average monthly income, the largest group comprised individuals with monthly incomes of NTD 20,000 or lower, and the second largest group had monthly incomes between NTD 20,001 and NTD 40,000; the two groups accounted for 40% and 15.8% of the respondents, respectively. In terms of the monthly budget that the respondents were willing to spend on gastrointestinal health foods, the largest group, which occupied 47.7% of the respondents, was willing to spend NTD 500 or lower; and in terms of monthly budget that the respondents were willing to spend on health promotion, the greater majority (83.4%) were willing to spend NTD 2000 or lower. For frequency of health checkups, more than half of the respondents (56.6%) were not in the habit of getting regular health checkups.

### 4.2. Reliability and Validity Analysis

Reliability was gauged using Cronbach’s α in this study to test internal consistency. Lee stated that an α value greater than 0.8 is optimal, whereas an α value equal to or greater than 0.7 and less than 0.8 indicates high reliability. The α values of all the constructs in this study were between 0.807 and 0.921, thereby indicating high internal consistency and fairly high reliability (Table 2).

Validity was examined using confirmatory factor analysis (CFA), and each construct was discussed. For content validity, the question items in this study were grounded on theories in existing literature and the questionnaire designs of various researchers, and they were then modified to fit our research field. Experts in marketing and food biotechnology evaluated and revised the question items, and further revisions were made after a pretest. Thus, the scale used in this study met the requirements for content validity.

Composite reliability and convergent validity were determined using composite reliability (CR) and average variance extracted (AVE) values. CR values greater than 0.6 indicate composite reliability, and AVE values greater than 0.5 indicate convergent validity [26]. The CR values in this study ranged from 0.757 to 0.905, and the AVE values ranged from 0.585 to 0.761 (Table 3). All were greater than the standard values. Thus, the results of this study had good composite reliability and convergent validity.

### 4.3. Analysis of Research Model Results

Table 4 displays the results of the overall model fit indices. The absolute fit indices were: χ^2^/*df* = 1.796, Goodness of Fit Index (GFI) = 0.932, Adjusted Goodness of Fit Index (AGFI) = 0.912, and Root Mean Square Error of Approximation (RMSEA) = 0.038; the incremental fit indices were: Non-Normed Fit Index (NNFI) = 0.980, Comparative Fit Index (CFI) = 0.982, and Incremental Fit Index (IFI) = 0.982; and the parsimony fit indices were Parsimony Normed Fit Index (PNFI) = 0.792, Parsimony Goodness of Fit Index (PGFI) = 0.712, and Critical N (CN) = 372.63. All of the indices met their respective criteria [24], thereby indicating a good model fit.

**Figure 3:**
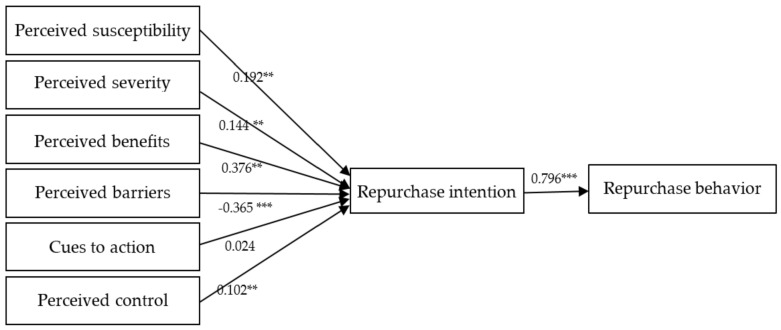
Structural equation modeling (SEM) analysis results. Note: ** *p* < 0.01, *** *p* < 0.001. The dashed line indicates that the influence was not statistically significant.

Table 5 presents the results of hypothesis testing. All of the path relationships in this study were significant, except for the result for H6, which examined the influence of cues to action on repurchase intention (Figure 3).

## 5. Discussions and Suggestions

No existing domestic studies applying the HBM combined with perceived behavioral control to health foods have focused on health foods that improve gastrointestinal functions. Those studies merely examined health foods as a broad category. This study therefore focused on a single health effect of health foods (that is, improving gastrointestinal functions) in order to provide a better picture of the actual consumption of health foods. Finally, we recommend the following actions for health food manufacturers and relevant authorities. We suggest the following: (1) Improving the pricing of health foods that improve gastrointestinal functions—our questionnaire revealed that most consumers are willing to spend NTD 500 or less per month on health foods that can improve gastrointestinal functions. We therefore suggest that health food manufacturers take this into account when setting the prices of their products. (2) Enhancing perceived susceptibility and severity of consumers regarding gastrointestinal diseases—the perceived susceptibility and perceived severity of individuals influence their repurchase intention toward health foods that improve gastrointestinal functions. When health food manufacturers are planning their marketing strategies for gastrointestinal health foods, they can enhance the perceived susceptibility and severity of gastrointestinal discomfort and relevant diseases so that consumers will perceive a need for gastrointestinal health foods and therefore be more likely to purchase them. (3) Enhancing perceived benefits and reducing perceived barriers of purchasing gastrointestinal health foods—the perceived benefits of action and perceived barriers of action of individuals influence their repurchase intention toward gastrointestinal health foods. When manufacturers are formulating marketing strategies and developing products involving gastrointestinal health foods, they should more strongly promote the benefits of their products for gastrointestinal functions. When consumers have doubts about barrier factors such as side effects, they should clarify and convey correct concepts. (4) Actively providing product information and catering to the purchasing habits of consumers—the perceived behavioral control of individuals influences their repurchase intention toward gastrointestinal health foods. When manufacturers are disseminating information on gastrointestinal health foods, they should educate consumers on relevant knowledge, nutrition, and certifications. In addition, they should make their products available through channels to which consumers are accustomed. (5) Promoting repurchase intention among consumers—the repurchase intention of individuals influence their repurchase behavior involving gastrointestinal health products. When manufacturers are planning marketing strategies for gastrointestinal health products, they should aim to promote the repurchase intention of consumers to develop repurchase behavior. (6) Addressing the lack of regular health checkups—our questionnaire revealed that over half of our respondents were not in the habit of getting regular health checkups. Less than 30% of the respondents had regular health checkups every year. Critically, achieving early prevention of gastrointestinal diseases requires regular checkups. The Health Promotion Administration of the Ministry of Health and Welfare [1] indicated that health checkups have been proven to detect colorectal cancer early and reduce its mortality rate. Indeed, regularly taking fecal occult blood tests every 1–2 years can reduce the colorectal cancer mortality rates of individuals between 50 and 69 years of age by 18–33%. With this, we suggest that relevant government departments devise ways to better and comprehensively implement regular health checkups.

## 6. Conclusions

Results from data analysis indicated that perceived susceptibility exerts a positive impact on repurchase intention. Regarding repurchase behavior involving gastrointestinal health foods, perceptions of possible gastrointestinal discomfort in individuals exert a positive influence on repurchase intentions. In other words, individuals with greater perceived susceptibility are more likely to have repurchase intentions toward health foods that improve gastrointestinal functions. This result is consistent with the findings of Rodriguez-Reimann et al. [11] in their study on the reasons behind the various tuberculosis prevention behaviors of Mexican Americans of different genders.

The first findings showed that perceived severity exerts a positive impact on repurchase intention. Regarding repurchase behavior involving gastrointestinal health foods, the subjective perceptions of gastrointestinal discomfort and the resulting medical and social consequences have a positive influence on repurchase intention. In other words, individuals with greater perceived severity are more likely to have repurchase intentions toward health foods that improve gastrointestinal functions, consistent with the results of a prior study of Chen and Tseng. Second, perceived benefits of action has a positive influence on repurchase intention. Regarding repurchase behavior involving gastrointestinal health foods, the subjective perception that purchasing gastrointestinal health foods will prevent functional gastrointestinal disorders or reduce the threat of gastrointestinal diseases has a positive impact on repurchase intention. In other words, individuals with greater perceived benefits of action are more likely to have repurchase intentions toward health foods that improve gastrointestinal functions. This result is consistent with the findings of Asare and Sharma [9] in their study on the sexual behavior and HIV/AIDS prevention measures of African immigrants in the US.

Next, perceived barriers of action exerts a negative impact on repurchase intention. Regarding repurchase behavior involving gastrointestinal health foods, the subjective perception of tangible and intangible obstacles that may be encountered while taking the action has a negative impact on repurchase intention. In other words, individuals with greater perceived barriers of action are less likely to have repurchase intentions toward health foods that improve gastrointestinal functions. This result is consistent with the findings of Feng et al. [27] in their study on the exercise behavior of university students in Taichung City. Further, perceived behavioral control exerts a positive impact on repurchase intention. Regarding repurchase behavior involving gastrointestinal health foods, the subjective perception of the difficulty perceived in purchasing gastrointestinal health foods based on self-efficacy and facilitating conditions exerts a positive influence on repurchase intention. In other words, individuals who perceive greater self-efficacy and better facilitating conditions are more likely to have repurchase intentions toward health foods that improve gastrointestinal functions. This result is consistent with the findings of Lin and Feng [17] in their investigation on factors that influence the behavioral intentions of adolescents regarding the consumption of tea-based drinks. In addition, repurchase intention exerts a positive influence on repurchase behavior. Regarding repurchase behavior involving gastrointestinal health foods, the possibility of an individual being willing to repurchase health foods that improve gastrointestinal functions has a positive influence on repurchase behavior. In other words, individuals with greater repurchase intention are more likely to display repurchase behavior regarding health foods that improve gastrointestinal functions. This result is consistent with the results of the purchase behavior model derived by Sheeran and Abraham [14].

Lastly, this study found that cues to action has insignificant influence on repurchase intention, although previous health-related studies supported that cues to action is a significant predictor for health behavior. One potential reason is that repurchase behavior involving gastrointestinal health foods and information sources that prompt individuals to purchase gastrointestinal health foods do not determine the public health promotion behavior, which is consistent with prior studies [2,26]. This study had some limitations concerning the selection of participations and adaptation of the constructs and their observed variables. Future studies could consider to include other variables and apply them to the general public.

## Figures and Tables

**Table 1:** Items of constructs.

Construct	Items	Source Literature
Perceived susceptibility	I might have gastrointestinal disorders (GID). My living habits might result in gastrointestinal disorders. The chance of having functional gastrointestinal disorders is higher than I expect. I feel unhealthy with my gastrointestinal disorders.	Rosenstock [2] Bish et al. [18]
Perceived severity	I am worried about gastrointestinal disorders. I think having gastrointestinal disorders is a serious problem. It will influence my family once I have gastrointestinal disorders. It will influence my work and social life once I have gastrointestinal disorders.	Rosenstock [2] Guvenc et al. [19]
Perceived benefits of action	Gastrointestinal health products could strengthen gastrointestinal functions. Gastrointestinal health products can effectively promote gastrointestinal health. Eating gastrointestinal health products that improve gastrointestinal functions is helpful to me.	Rosenstock [2] Chen et al. [20]
Perceived barriers of action	I usually disagree with using health foods. Health foods will cause side effects. Eating health foods is inconvenient to me. Health foods associate with negative things to me.	Rosenstock [2] Chen et al. [20]
Cues to action	When purchasing health foods, I will refer to the opinions of my family. When purchasing health foods, I will refer to the opinions of my friends. When purchasing health foods, I will refer to the opinions of my colleagues/classmates. When purchasing health foods, I will try my best to meet the suggestions and expectations of people around me. When purchasing health foods, I will refer to the opinions of government agencies (e.g., Food and Drug Administration). When purchasing health foods, I will refer to the opinions of the media (e.g., television, newspaper and magazines, social networks).	Rosenstock [2] Schiffman and Kanuk [21]
Perceived behavioral control	I have the ability to judge the certification labels of health foods. I have the ability to understand the nutritional content of health foods. I have enough knowledge about purchasing health food products that I want. I have enough time to choose and purchase the health food products that I want. I have enough channels and approaches to purchase the health food products that I want.	Ajzen [22] Taylor and Todd [15]
Repurchase intention	I have plans to purchase health food products. I will consider purchasing health food in the immediate future. I am willing to buy health food products. I will recommend others to buy health food products.	Dodds and Monroe [23]
Repurchase behavior	I have been frequently eating health foods recently. I spend a part of my living budget on purchasing health food products. I often purchase health food products for my own use. I often purchase health food products for my family to use.	Schiffman and Kanuk [21]

**Table 2:** Cronbach’s α of constructs.

Construct	Cronbach’s α	Construct	Cronbach’s α
Perceived susceptibility	0.921	Cues to action	0.851
Perceived severity	0.821	Perceived behavioral control	0.902
Perceived benefits of action	0.906	Repurchase intention	0.848
Perceived barriers of action	0.807	Repurchase behavior	0.920

**Table 3:** Composite reliability (CR) and average variance extracted (AVE) values of constructs.

Latent Variable	CR	AVE	Latent Variable	CR	AVE
Perceived susceptibility	0.872	0.695	Cues to action	0.875	0.707
Perceived severity	0.829	0.621	Perceived behavioral control	0.905	0.761
Perceived benefits of action	0.757	0.609	Repurchase intention	0.798	0.664
Perceived barriers of action	0.808	0.585	Repurchase behavior	0.862	0.757

**Table 4:** Goodness-of-fit indices of structural equation model.

Test Statistic		Criterion	Value in This Study
Absolute fit indices	χ^2^		515.731
	d.f.		287
	χ^2^/d.f.	<5	1.796
	GFI	≥0.8	0.932
	AGFI	>0.8	0.912
	RMSEA	<0.08	0.038
Incremental fit indices	NNFI	≥0.9	0.980
	CFI	≥0.9	0.982
	IFI	>0.9	0.982
Parsimony fit indices	PNFI	≥0.5	0.792
	PGFI	≥0.5	0.712
	CN	>200	372.63

**Table 5:** Path coefficients and hypothesis testing results.

Path	Expected Sign	Path Coefficient	Test Result
H1: Perceived susceptibility → repurchase intention	+	0.192 **	Supported
H2: Perceived severity → repurchase intention	+	0.144 **	Supported
H3: Perceived benefits of action → repurchase intention	+	0.376 ***	Supported
H4: Perceived barriers of action → repurchase intention	−	−0.365 ***	Supported
H5: Cues to action → repurchase intention	+	0.024	Not supported
H6: Perceived behavioral control → repurchase intention	+	0.102 **	Supported
H7: Repurchase intention → repurchase behavior	+	0.796 ***	Supported

Note: ** *p* < 0.01, *** *p* < 0.001.
